# Awareness of family health history in a predominantly young adult population

**DOI:** 10.1371/journal.pone.0224283

**Published:** 2019-10-25

**Authors:** Sarina Madhavan, Emily Bullis, Rachel Myers, Chris J. Zhou, Elise M. Cai, Anu Sharma, Shreya Bhatia, Lori A. Orlando, Susanne B. Haga

**Affiliations:** 1 Duke University, Trinity Arts and Sciences, Durham, North Carolina, United States of America; 2 Duke University, Initiative for Society and Society, Durham, North Carolina, United States of America; 3 Center for Applied Genomics and Precision Medicine, Duke University School of Medicine, Durham, North Carolina, United States of America; 4 Duke University, Pratt School of Engineering, Durham, North Carolina, United States of America; Aga Khan University, KENYA

## Abstract

Family health history (FHH) is a key predictor of health risk and is universally important in preventive care. However, patients may not be aware of the importance of FHH, and thus, may fail to accurately or completely share FHH with health providers, thereby limiting its utility. In this study, we conducted an online survey of 294 young adults and employees based at a US university setting regarding their knowledge, sharing behaviors, and perceived importance of FHH, and use of electronic clinical tools to document and update FHH. We also evaluated two educational interventions (written and video) to promote knowledge about FHH and its importance to health. We found that 93% of respondents were highly aware of their FHH, though only 39% reported collecting it and 4% using an online FHH tool. Seventy-three percent of respondents, particularly women, had shared FHH with their doctor when prompted, and fewer had shared it with family members. Participants in the video group were significantly more likely to understand the benefits of FHH than those in the written group (p = 0.02). In summary, educational resources, either video or written, will be helpful to promote FHH collection, sharing, and use of online FHH tools.

## Introduction

Family health history (FHH) is a strong predictor of disease risk and useful for guiding preventive care, yet is persistently underutilized in clinical care. Barriers include patient’s limited knowledge about their FHH, the length of time it takes to collect a full FHH at the point of care, lack of awareness of the benefits of a FHH-based risk assessment, and the complexity of synthesizing the data into and actionable care plans [1–4]. In addition, Public understanding of the clinical significance of FHH varies [5, 6], and other factors such as family dynamics[7], culture[8], privacy[9], and openness about family members’ health impact completeness of the information patients share with their providers. Its predictive value pivots on the quantity and quality of FHH information collected from the patient, and its clinical utility on whether providers adopt appropriate guideline-based action(s). In order to fully realize its benefits, a family health history-based risk assessment should ideally be initiated as a young adult, prior to disease onset.

While FHH is still commonly collected on paper-based patient intake forms during appointment check-in and then reviewed by a health provider, several online tools have been developed to facilitate the collection and interpretation of FHH [10]. For example, a group of investigators at Duke has developed a FHH clinical decision support system (CDSS) in primary care practices called MeTree [11, 12], and evaluated its clinical utility as part of one of the first programs supported by the NIH’s **I**mplementing **G**e**N**omics **I**n Prac**T**ic**E** (IGNITE). MeTree facilitates collection of a three-plus generation FHH for 128 medical diseases, generates evidence-based recommendations for both patients and providers for 45 diseases, and enables convenient updating and visualization of FHH [11]. It also enables families to collaborate on data entry and sharing of risk information. Although MeTree and other CDSS tools have advanced FHH collection, issues of e-health literacy and the aforementioned challenges are still relevant to patient utilization of digital-based FHH tools. Thus, the combination of a high-quality engagement strategy, a user-friendly application, and patient educational materials are likely needed to maximize utilization of tools to improve risk assessment and risk management in primary and specialty care.

Since risk assessment is ideally performed before age 30 to initiate appropriate risk prevention prior to disease onset, raising awareness of FHH in younger patient populations is an important goal. Knowledge about young adults’ comprehension and attitudes toward FHH is limited. Smith et al. [13] surveyed a cohort of college students and reported that the principal driver behind FHH collection or learning was the development of a hereditary condition in a family member or themselves. The study also suggested that many (56%) of college student participants have yet to discuss FHH with their physician, yet most (81%) would be willing to at some point.

In this study, we assessed young adults’ attitudes and knowledge about FHH to begin to understand the barriers to FHH-based risk assessment in young adults. In addition, we developed and evaluated educational resources designed specifically for young adults. These included a narrative video and text-based patient materials about why and how to collect FHH from relatives, hypothesizing that a video may be more effective for younger populations. Videos can be a highly effective method to provide guidance and instructions [14], facilitate informed decision-making [15], and encourage adoption of healthy behaviors [16, 17], and be more effective than written informational materials in some instances [18]. Our findings will help inform development of educational resources to promote patient understanding of the importance of FHH.

## Materials and methods

### Survey experiment and design

We conducted an online survey to assess knowledge about the importance of FHH, experience with FHH, and knowledge of family members’ health history in a primarily young adult population. Basic demographic information was also collected. In addition, participants were randomized to evaluate one of the two types of educational resources about FHH and MeTree. The study was approved by the Institutional Review Board of Duke University (#2018–0277).

### Study population and recruitment

Participants were recruited across Duke University through multiple types of outreach and advertising efforts, including housing listserv emails, social media (Facebook), person-to-person contact, and flyers posted around the Duke campus. This was an “open survey” and the study recruitment flyers directed prospective participants to a URL for the home (landing) page of the survey. The landing page included information about the purpose of the study, risks and benefits, approximate length of time to participate in the survey (45–60 minutes), and a reminder that participation in this study is both anonymous and voluntary (participants can exit the survey at any point). Participation in the study was incentivized through a drawing for a $100 Visa gift card. We do not have any estimates regarding the total number of employees, faculty, trainees, and students on the main campus and thus, were not able to calculate a response rate.

### Survey tool

The survey focused on behaviors, attitudes, and perceived utility of FHH and FHH collection. The survey was comprised of 38 questions and divided into five major sections: demographics, familiarity with FHH and knowledge of and experiences with their personal FHH, interest/attitudes toward FHH, attitudes toward MeTree specifically, and a discrete choice experiment (excluded from data reported here) (see S1 Text). Demographic questions included age group, gender, occupation (if not a student), major, and year in degree program for student respondents. We examined other FHH surveys when phrasing knowledge and attitudinal questions [19–24]. Familiarity with FHH was assessed by familiarity with the following phrases: family health history, hereditary cancer risk, hereditary disease risk, and genetic testing. No personal health information was collected in the survey; contact information was collected for entry into the drawing for the gift card through a separate and not linked to the participant’s responses. All data were stored in a password-protected university-based network. The survey questions and answer responses were reviewed by peers of the study team and revised accordingly.

### Design of educational materials

Three educational videos were developed to provide an overview of both FHH and MeTree for all educational levels. The script was adapted from an educational worksheet originally developed for MeTree and a CDC survey [20] (see S2 Text). We employed message-framing strategies recommended for patient health education resources [25, 26], including phrasing recommendations as positively-framed actions, conveying primary recommendations via physician authority, and modeling behaviors from family members and fictional MeTree end-users to increase viewers’ self-efficacy [27]. The videos also included FHH strategies for when and how to acquire FHH, a suggested list of questions to ask family members, and answers to common concerns about FHH collection and sharing. Through a vignette, we also modeled a FHH discussion between a mother and son. The videos were produced through Duke Media Services and filmed on-location (Duke clinics) and at staged filming areas. The video lengths were 2:49, 7:05, and 3:12 for a combined total of 13:06 minutes. Actors (with the exception of the physician) were recruited to represent diverse backgrounds. Our combined young adult (student) and faculty team informed the development of the scripts and scenarios that aimed to be appealing and understandable to a younger population, who were likely to be unfamiliar with FHH, while accurate and comprehensive. The printed materials were developed by the MeTree team and used in a prior study [28]. S1 Table compares the key features of the MeTree worksheet and the narrative video developed for this study. There were several differences with respect to scope and depth of content and word count (the video was 25% longer than the written material), the reading level was similar (8^th^ vs. 9^th^ Flesch-Kincaid grade).

Upon acknowledgement that they have read the information about the study and their rights as participants (by checking a box at the bottom of page), participants were randomly assigned to either view the standard written informational materials that were originally provided to MeTree participants in previous studies [28] or to view a newly developed narrative educational video.

### Analysis

Participant demographics, survey response rates, and survey responses were summarized using either counts and percentages or mean, median, and standard deviation in all participants, as well as, stratified by participants who did and did not complete the study. Assessments of independence between completion of the survey and the participants’ demographics or survey responses were assessed using Pearson’s chi-squared tests or Wilcoxon rank sum tests. Among the participants who completed the study, Pearson’s chi-squared tests or Wilcoxon rank sum tests were used to test for non-independence between survey responses and participant demographics. The significance threshold for all tests was 0.05, with no adjustments for multiple testing. All statistical analyses were conducted using R software (v3.4.0). To analyze responses to the open-ended questions, all authors coded the responses and analyzed them with NVivo 11 software. An inductive content analysis was performed to identify trends in responses and common themes around barriers to collecting FHH.

## Results

### Participant demographics

We received a total of 294 completed survey responses; another 213 respondents partially completed the surveys. The majority of respondents were Duke undergraduates (55%), female (67%), aged 18–29 years (82%; here forth, referred to as ‘younger adults’ in this paper) (Table 1). We captured a relatively even representation from each year in both the undergraduate (freshman, sophomore, etc.) and graduate student population (1st year, 2nd year, etc.). Within both the Duke graduate and undergraduate populations, science, engineering, and mathematics majors were dominant, comprising 72% of the undergraduate student population and 53% of the graduate student population.

**Table 1:** Participant characteristics.

Characteristics	No. (n = 294) (%)
Gender	
• Male	96 (33%)
• Female	197 (67%)
• Other	1 (0.34%)
Age	
• 18–29 years	240 (82%)
• ≥30 years	54 (18%)
Duke Affiliation	
• Undergraduate Student	162 (55%)
• Graduate Student	61 (21%)
• Staff	37 (12%)
Other	34 (12%)
• Education (Non-students)	
• Less than high school	2 (3%)
• High School	61 (21%)
• Some college or 2-year degree	4 (6%)
• 4-year college degree	24 (34%)
• Post-graduate degree	32 (46%)
Undergraduate Year	
• Freshman	35 (22%)
• Sophomore	56 (35%)
• Junior	34 (21%)
• Senior	36 (22%)

*For undergraduate program of study, more than one answer could be selected.

We did not observe any differences between participants who completed and did not complete the survey with respect to gender, age group, occupation, and majors (among undergraduate and graduate student participants). Differences by education level were observed, with participants with higher levels of education more likely to have completed the survey, possibly due to the complexity of the subject matter (further supported by observation of dropout at the beginning of the section on FHH knowledge and behaviors). We also examined completion by family history of cancer, cardiovascular disease, and diabetes and found evidence of non-independences between those responses and completion as well as differences between FHH sharing behaviors (those who completed the survey were more likely to report sharing of FHH information).

### Findings from the online survey

#### Familiarity with & collection of FHH

Ninety-three percent of respondents had heard of the phrase ‘family health history’, 78% had heard of ‘hereditary cancer risk’, 77% had heard of ‘hereditary disease risk’, and 89% had heard of ‘genetic testing’. The majority (68%) were familiar with all four phrases (2% had heard of none of the phrases). Pre-medical students had significantly greater background knowledge of phrases associated with FHH than other majors (Wilcox rank sum test p = 0.0002).

Overall, 39% had collected FHH. One third (31%) of pre-medical students had collected their FHH compared to 41% of non-pre-medical students (chi-square test p value = 0.28) and women were significantly more likely to have collected their FHH (chi-square p value = 0.045). Twenty-one percent indicated they have a record (written or electronic) of their FHH, with a larger proportion of younger participants (age 18–29 yo) and women having a record of their FHH (chi-square test p value = 0.034, 0.047, respectively). Only 4% of respondents had used an online FHH tool to determine disease risk.

#### FHH sharing behaviors

A total of 73% of respondents indicated they have shared FHH with their doctor when asked, with family members (35%), or with other individuals (19%). Respondents who had relatives with cancer or heart disease were more likely to have shared FHH with family members than those without relatives with cancer or heart disease (chi square p values = 0.026, and 0.013, respectively), but were not more likely to share with their doctor. Women were more likely to share with their doctor when prompted (chi square p value < 0.001) and men were significantly more likely to not share their FHH with anyone (chi square p value = 0.004). Age group was not associated with sharing FHH with a doctor.

### Findings from interventional study

#### FHH collection & sharing

**Fig 1:**
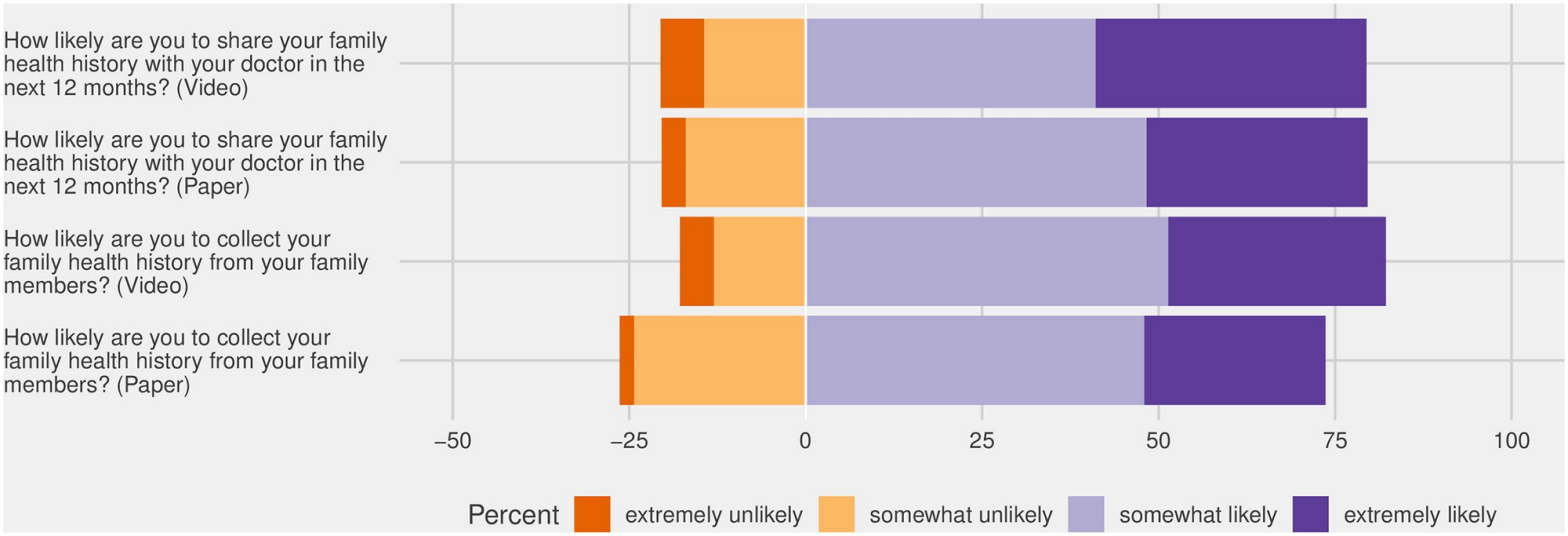
Likelihood of FHH collection and sharing post- educational intervention.

After reviewing the educational materials (either the written MeTree resources (n = 148) or the video (n = 146)), respondents were asked about their attitudes regarding FHH collection and sharing (Fig 1). The only difference we observed between the study groups was that video participants trended towards reporting that they would be extremely or somewhat likely to collect FHH from family members (p = 0.056). No difference by age or gender was observed with respect to collecting FHH.

Regarding likelihood to share FHH with a doctor in the coming year, we observed no differences by educational intervention group and all other demographic features, except among participants with a first degree relative with diabetes–those with a first degree relative were more likely to share FHH with their doctor (chi square p value = 0.004).

#### Attitudes toward FHH

**Fig 2:**
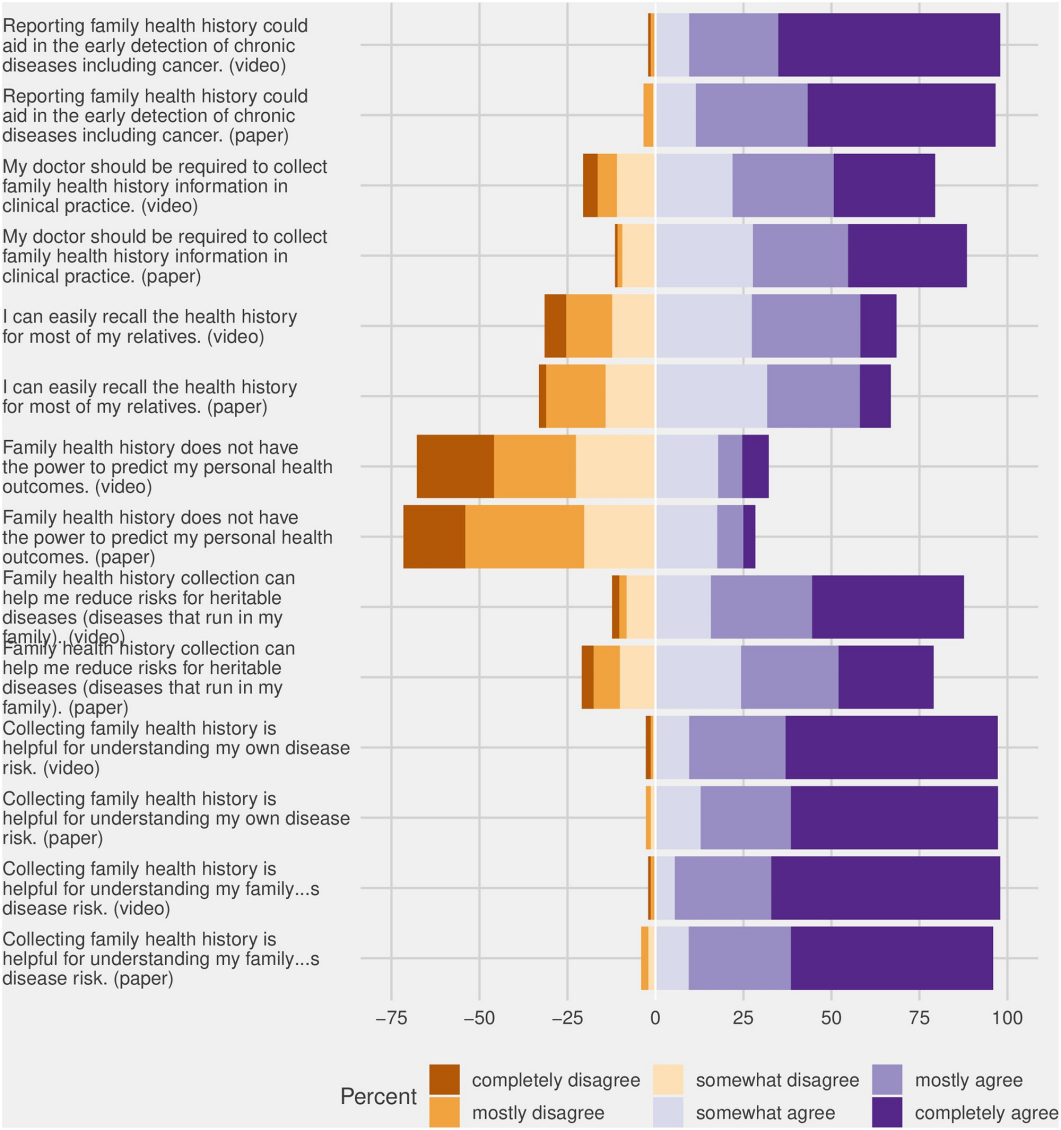
Attitudes towards FHH and perceived value (Video (V); n = 146); written material (MeTree); n = 148).

Overall, 60% of respondents completely agreed with the statement that collecting FHH is helpful for understanding their own disease risk and 61.5% completely agreed with the statement that collecting FHH is helpful for understanding their family’s disease risk (Fig 2). Thirty-five percent completely agreed with the statement that FHH collection can help reduce risks for hereditary diseases and 58% completely agreed that reporting FHH could aid in the early detection of chronic diseases including cancer. Older adults tended to have more extreme attitudes (i.e. completely agree/disagree) regarding the power FHH has to predict personal health outcomes (chi square test p value = 0.002), however both groups had similar proportions of overall agreement with the power of FHH to predict disease risk.

Participants in the video study group were significantly more likely to agree with the statement “Family health history collection can help me reduce risks for hereditary diseases” than those in the written resources group (chi square p value = 0.02). No other differences in attitudes between intervention groups was observed.

In response to an open-ended question regarding barriers to collecting FHH, three major reasons were indicated: family dynamics (29%), time required to collect FHH (23%), and incomplete or insufficient FHH records (20%).

#### Attitudes toward MeTree

**Fig 3:**
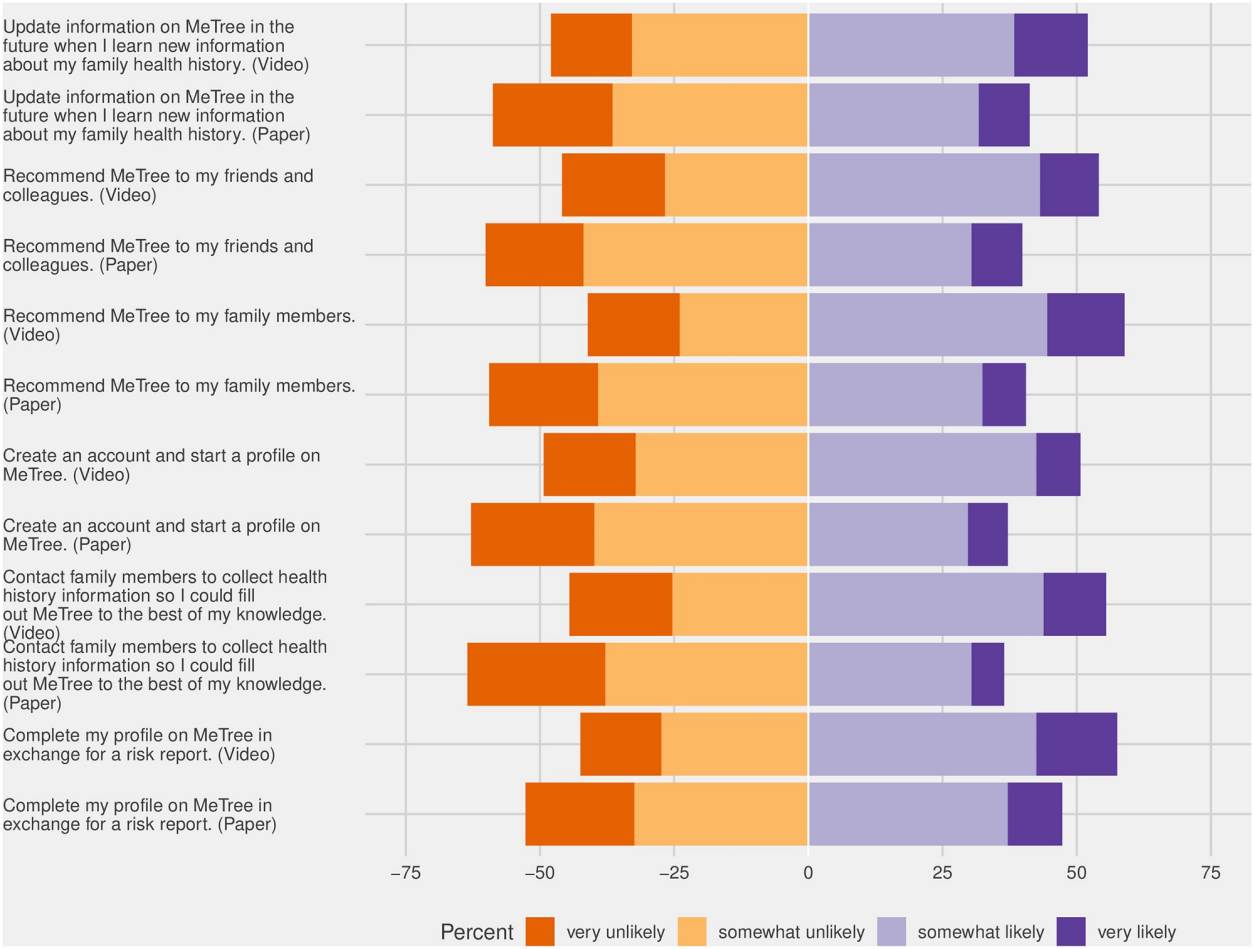
Attitudes towards MeTree (V = video arm; MeTree = MeTree written materials arm).

After a description of MeTree was provided to respondents in both groups, we asked respondents to indicate their likelihood of using MeTree (Fig 3). Overall, 22% would be very comfortable with sharing personal health data with MeTree, compared to 7% who would be very uncomfortable. Respondents randomized to the video arm were significantly more likely to indicate they would contact family members about their FHH in order to complete MeTree (p = 0.01); to recommend MeTree to family members (p = 0.01), or friends and colleagues (p = 0.04). Respondents randomized to the video group were also more likely to cite higher levels of comfort with entering FHH into MeTree (p < 0.001).

Overall, older adults were significantly more likely than younger adults to indicate an interest in creating an account and starting a profile on MeTree (p = 0.03); completing a profile on MeTree in exchange for a risk report (p = 0.009); and updating MeTree upon learning new information about their personal FHH (p = 0.04). Respondents with a high school diploma or less reported lower interest in creating or filling out a MeTree profile (p = 0.04). Individuals with some college education or more were more likely to cite family dynamics (p = 0.003) and inconsistent or inadequate healthcare lowering their perceived value of FHH (p = 0.01) as barriers to providing FHH.

In the open-ended response question regarding barriers to using MeTree, male respondents were more likely than female respondents to cite mistrust of MeTree or privacy concerns (p = 0.02). Females were less likely than males to cite anxieties about the results report that MeTree generates (p = 0.02) and family size as impeding FHH collection (p = 0.01).

## Discussion

This study aimed to gain insight about knowledge and attitudes of FHH in an understudied group, mostly healthy young adults (18–29 yo); and to evaluate two types of educational resources designed to improve awareness and perceived value of FHH and the availability of collection tools like MeTree. While there was high familiarity with FHH and inherited diseases, the majority of respondents did not actively gather FHH from their family members nor have any written or electronic record of FHH, similar to findings in other studies [29]. The narrative, educational video format was more effective at increasing comfortability, attitudes and perceived utility of FHH and MeTree as a risk assessment and prevention tool compared to a more traditional written resource with similar content.

This study suggests that certain factors like proximity to certain disease (e.g., participants with relatives with heart disease or cancer) is associated with sharing FHH with family members, but not necessarily doctors, suggesting that the significance of FHH may be unclear. Alternatively, some individuals may believe that no action can be taken to reduce or prevent disease onset if it runs in the family (fatalistic) [30, 31]. Our findings also suggest that age may be an important factor for perceived utility of FHH, with the older study population being more proactive in collecting and reporting FHH and more likely to use an online tool like MeTree. Older individuals may feel a higher sense of relevancy and urgency in collecting health information than younger individuals. In addition, older members of families (>50 years old) often serve as historians of FHH and therefore, have higher levels of knowledge about FHH compared to other age groups [19]. Other demographic data demonstrated that men responded as having more mistrust of MeTree than women. Yet women were more likely to share FHH with their doctor when prompted, reflective of higher number of ambulatory care visits [32] and utilization of health services [33–36]. At baseline pre-medical (as compared to non pre-medical) students were more knowledgeable of FHH-related terms; however, there were no differences in attitudes towards FHH collection.

Online health tools like MeTree can be valuable to both patients and clinicians to optimize FHH data collection, analysis/interpretation, and care. However, the benefits of such applications will depend on patient utilization, which in turn will be impacted by their knowledge of FHH, perceived value, and self-efficacy. The MeTree team has developed background materials for patients prior to completing their online MeTree profile [37], as well as added pop-up graphics with tips and instructions, and pop-up content from MedlinePlus Connect (https://medlineplus.gov/connect/overview.html) written at a 5th grade reading level for context-sensitive help. Tools and resources to increase understanding of the significance of FHH can increase sharing and collecting FHH between family members [38].

Several limitations of the study should be noted. Individuals with some college or greater level of education represented the majority of the survey respondents. Furthermore, a large proportion of the students (both undergraduate and graduate) were studying in a STEM discipline. Because science majors and university students are likely to be more familiar with scientific terminology and more health literate, results from this study may have been skewed. However, it should be noted that pre-medical or belonging to a STEM major was not a predictor of perceived utility of FHH, but rather was merely a predictor of familiarity with FHH terminology. Future research should seek to recruit a more varied demographic to assess attitudes and utility of educational video formats for improving all patient attitudes and engagement with FHH. Furthermore, we did not collect race, ethnicity, nor SES data and therefore, our findings are limited in understanding the complicated relationship between certain demographic factors and FHH attitudes. Further investigation may be warranted to better characterize participants who did not complete the survey compared to how they differ from participants who completed the survey, and what we can learn about participants who drop out of research studies.

Certain FHH collection behaviors, common misconceptions and unfamiliarity can be addressed with greater public awareness about the significance of FHH. In addition, more support is likely needed to help patients navigate different family structures and dynamics, and sharing of health information about diseases and health histories associated with stigma, denial and embarrassment. As families may utilize different communication patterns, future interventions should be sensitive to cultural traditions, gender and generational differences, and literacy. Disclosure of information about the Genetic Information Nondiscrimination Act (GINA) could address patient concerns regarding confidentiality and privacy. Lastly, since the young adult population can act as effective communicators in families faced with language and geographic barriers, future interventions should target self-efficacy in the young adult population. By promoting the importance of FHH to the ‘keepers’ of valuable FHH information (the older generation), the younger generation can serve as a bridge. Ultimately, providing effective educational tools such as videos and narratives that elucidate the value of FHH to patients while also supplying them with skills necessary to optimize collection and sharing of FHH are imperative for improving precision medicine outcomes.

## Supporting information

S1 Text Survey instrument. (DOCX)

S2 Text Video scripts and written MeTree materials. (DOCX)

S1 Table A comparison of the features of the video and written MeTree educational materials. (DOCX)

S1 Dataset Raw survey data file. (XLSX)
