# Supplementary material for: Awareness of family health history in a predominantly young adult population
Source: PLoS One. 2019 Oct 25;14(10):e0224283. doi: 10.1371/journal.pone.0224283 (PMC6814221; doi:10.1371/journal.pone.0224283)
Supplement: S1 Text — (DOCX) [file pone.0224283.s001.docx]

**S1 Text. Full Survey**:

**Enabling PM Bass Connections: FHH/MeTree (Full Survey)**

**Start of Block: Consent**

|  |
| --- |

Q1.1 Welcome and thank you for your interest in our study!  This study is being conducted by the Bass Connections team: Enabling Precision Medicine and Health- a research group at Duke University. This research team consists of students and faculty who want to examine how people view their family health history and whether or not providing informational materials before health history collection could help patients understand its benefit in managing disease risk.By participating in this survey you will be asked to first watch/read an informational resource describing important information about family health history. Afterward, you will be asked to complete a survey about family health history. This entire study should take no longer than 30 minutes all together to complete.

Data Collection:Your participation in this study is completely anonymous. Your email will never be attached to your survey responses; we do not ask for your name or any other information that might identify you personally.

Participation:Additionally, your participation in this research study is completely voluntary. You may withdraw at any time and you may choose not to answer any question.

Compensation:After completing the survey, you will be given the option to provide us with your email address and contact information. Your email and contact information will be collected completely separate from your survey responses and will be included in a random raffle drawing for the opportunity to receive one of four $100 MasterCard/Visa gift cards. If you are a raffle winner, we will email you to arrange how to pick up your $100 MasterCard/Visa gift card. You will also be required to provide your SSN or DUID if you are a raffle winner.

Contact Info: If you have any questions about this study, please contact fhhbass@gmail.com or Dr. Susanne Haga at susanne.haga@duke.edu. For questions about your rights as a participant contact the Duke IRB at campusirb@duke.edu or (919)-684-3030.

| Page Break |  |
| --- | --- |

**End of Block: Consent**

**Start of Block: Demographics**

|  |
| --- |

Q2.1 What is your gender?

- Male  (1)
- Female  (2)
- Other  (3)

|  |
| --- |

Q2.2 What is your occupation?

- Undergraduate student  (1)
- Graduate student  (2)
- Duke University staff  (3)
- Other  (4) ________________________________________________

*Display This Question:*

*If What is your occupation? = Undergraduate student*

|  |
| --- |

Q2.3 Select your year

- Freshman  (1)
- Sophomore  (2)
- Junior  (3)
- Senior  (4)
- Other (please specify)  (5) ________________________________________________

*Display This Question:*

*If What is your occupation? = Undergraduate student*

|  |
| --- |

Q2.4 Select program of study (select all that apply)

- Natural Sciences (Physics, Chemistry, Biology, Environmental science, etc.)  (1)
- Engineering  (2)
- Math and Computer Science  (3)
- Languages and Humanities (English, Philosophy)  (4)
- Social Science (Sociology, Public Policy, Political Science, Psychology, Economics)  (5)
- Other  (6)

*Display This Question:*

*If What is your occupation? = Undergraduate student*

|  |
| --- |

Q2.5 Are you a pre-med student?

- Yes  (1)
- No  (2)

*Display This Question:*

*If What is your occupation? = Graduate student*

|  |
| --- |

Q2.6 Area of graduate studies (choose all that apply)

- Natural Sciences (Physics, Chemistry, Biology, Environmental science, etc.)  (1)
- Engineering  (2)
- Math and Computer Science  (3)
- Languages and Humanities (English, Philosophy)  (4)
- Social Science (Sociology, Public Policy, Political Science, Psychology, Economics)  (5)
- Medicine  (6)
- Law  (7)
- Business  (8)
- Other: Please specify  (9) ________________________________________________

*Display This Question:*

*If What is your occupation? = Graduate student*

|  |
| --- |

Q2.7 Year in Program

- 1 Year  (1)
- 2 Years  (2)
- 3-4 Years  (3)
- 5+ Years  (4)

*Display This Question:*

*If What is your occupation? = Duke University staff*

*Or What is your occupation? = Other*

|  |
| --- |

Q2.8 Highest education

- Less than high school  (1)
- High school graduate or GED  (2)
- Trade or technical school  (3)
- Some college or 2-year degree  (4)
- 4-year college graduate  (5)
- Post-graduate degree  (6)

|  |
| --- |

Q2.9 What is your age range?

- younger than 18  (1)
- 18-29 years old  (2)
- 30 or older  (3)

*Display This Question:*

*If What is your age range? = younger than 18*

|  |
| --- |

Q38 We appreciate your enthusiasm in participating in this research. Unfortunately, the minimum age required to participate is 18. You will now be directed to the end of the survey.

*Skip To: End of Survey If We appreciate your enthusiasm in participating in this research. Unfortunately, the minimum age r...() Is Displayed*

**End of Block: Demographics**

**Start of Block: Health background and attitudes**

|  |
| --- |

Q3.1 Have any of your 1st degree relatives (your birth mother, birth father, brother(s), sister(s), son(s), daughter(s)) been diagnosed with the following?

|  | Yes (1) | No (2) | Unknown (3) | Prefer Not to Answer (4) |
| --- | --- | --- | --- | --- |
| Cancer (any type) (1) |  | • | • | • |
| Heart and cardiovascular diseases (high blood pressure, irregular heartbeat, etc.) (2) |  | • | • | • |
| Diabetes (3) |  | • | • | • |

|  |
| --- |

Q3.2 Have any of your 2nd degree relatives (aunts, uncles, grandparents, grandchildren, nieces, nephews, or half-siblings) been diagnosed with the following?

|  | Yes (1) | No (2) | Unknown (3) | Prefer Not to Answer (4) |
| --- | --- | --- | --- | --- |
| Cancer (any type) (1) |  | • | • | • |
| Heart and cardiovascular diseases (high blood pressure, irregular heartbeat, etc.) (2) |  | • | • | • |
| Diabetes (3) |  | • | • | • |

|  |
| --- |

Q3.3 Do any other diseases run in the family?

- Yes (please specify)  (1) ________________________________________________
- No  (2)
- Unknown  (3)
- Prefer Not to Answer  (4)

|  |
| --- |

Q3.4 Have you been diagnosed with the following?

|  | Yes (1) | No (2) | Unknown (3) | Prefer Not to Answer (4) |
| --- | --- | --- | --- | --- |
| Cancer (any type) (1) |  | • | • | • |
| Heart and cardiovascular diseases (high blood pressure, irregular heartbeat, etc.) (2) |  | • | • | • |
| Diabetes (3) |  | • | • | • |

|  |
| --- |

Q3.5 Do you have any other diseases?

- Yes (please specify)  (1) ________________________________________________
- No  (2)
- Unknown  (3)
- Prefer Not to Answer  (4)

|  |
| --- |

Q4.1 Many people are not familiar with the concept of family health history and terms related to family health history. Have you heard of any of these terms before? Select all terms that you have heard of at any point before viewing today’s materials (select all that apply):

- Family health history  (1)
- Hereditary cancer risk  (2)
- Hereditary disease risk  (3)
- Genetic testing  (4)
- I have not heard of any of the above  (5)

|  |
| --- |

Q4.2 Have you collected your family health history? For example: asking distant family members about their health, finding patterns in disease that runs in the family, etc.)

- Yes  (1)
- No  (2)

|  |
| --- |

Q4.4 Do you have a record (written or electronic) of your family health history?

- Yes  (1)
- No  (2)

|  |
| --- |

Q4.5 Have you reported your family health history to your doctor?

- Yes  (1)
- No  (2)

|  |
| --- |

Q4.7 Have you used an online family health history tool to determine your risk of disease?

- Yes  (1)
- No  (2)

|  |
| --- |

Q4.8 Before taking this survey, have you shared your family health history before (e.g., a list of diseases that run in your family like diabetes; and/or family member’s lifestyle behaviors like cigarette smoking), in any of the following ways? (select all that apply)

- My doctor asked about my family health history, so I discussed it with him/her.   (1)
- I shared my family health history with my doctor without him/her asking.   (2)
- I have shared my family health history with family members.  (3)
- I have posted my family health history on social media (for example Facebook, Twitter, etc).  (4)
- I have shared my family health history with someone other than a doctor or family member.  (5)
- I have not shared this information with anyone.  (6)
- I don’t know anything about my family health history.  (7)

**End of Block: Health background and attitudes**

**Start of Block: Videos**

|  |
| --- |

Q63 Please view the following videos before continuing. Be sure to view all 3 *without* skipping through. We appreciate your cooperation.

|  |
| --- |

Q63

|  |
| --- |

Q58 - vid time Timing

First Click  (1)

Last Click  (2)

Page Submit  (3)

Click Count  (4)

**End of Block: Videos**

**Start of Block: Written Materials**

|  |
| --- |

Q63
Please open this link in a new tab and read the document carefully. The process is critical to the purpose of our survey, and we appreciate your patience. When you are finished, please return to this survey and continue. You may leave the worksheet open and refer to it while completing the survey.Metree website instructions worksheet

|  |
| --- |

Q64 - written time Timing

First Click  (1)

Last Click  (2)

Page Submit  (3)

Click Count  (4)

**End of Block: Written Materials**

**Start of Block: Perceptions of FHH Utility**

|  |
| --- |

Q4.3 How likely are you to collect your family health history from your family members?

- Extremely likely  (1)
- Somewhat likely  (2)
- Somewhat unlikely  (3)
- Extremely unlikely  (4)

|  |
| --- |

Q4.6 How likely are you to share your family health history with your doctor in the next 12 months?

- Extremely likely  (1)
- Somewhat likely  (2)
- Somewhat unlikely  (3)
- Extremely unlikely  (4)

|  |
| --- |

Q4.9 For the following questions, please rate your response on a scale from 1 to 6, with breakdown as follows.

|  | Completely Disagree (1) | Mostly Disagree (2) | Somewhat Disagree (3) | Somewhat Agree (4) | Mostly Agree (5) | Completely Agree (6) |
| --- | --- | --- | --- | --- | --- | --- |
| I can easily recall the health history for most of my relatives. (1) |  | • | • | • | • | • |
| Collecting family health history is helpful for understanding my own disease risk. (2) |  | • | • | • | • | • |
| Collecting family health history is helpful for understanding my family’s disease risk. (3) |  | • | • | • | • | • |
| Family health history collection can help me reduce risks for heritable diseases (diseases that run in my family). (4) |  | • | • | • | • | • |
| Reporting family health history could aid in the early detection of chronic diseases including cancer. (5) |  | • | • | • | • | • |
| My doctor should be required to collect family health history information in clinical practice. (6) |  | • | • | • | • | • |
| Family health history does not have the power to predict my personal health outcomes. (7) |  | • | • | • | • | • |

**End of Block: Perceptions of FHH Utility**

**Start of Block: MeTree Evaluation**

|  |
| --- |

Q5.1 Evaluation of MeTreeMeTree is an online tool that helps you keep track of your personal and family health history. It also gives you personalized recommendations based on your risk for 30 chronic diseases, including cancer. It is created by a team of researchers at Duke Health. Completing MeTree online would look something like this:

|  |
| --- |

Q5.2


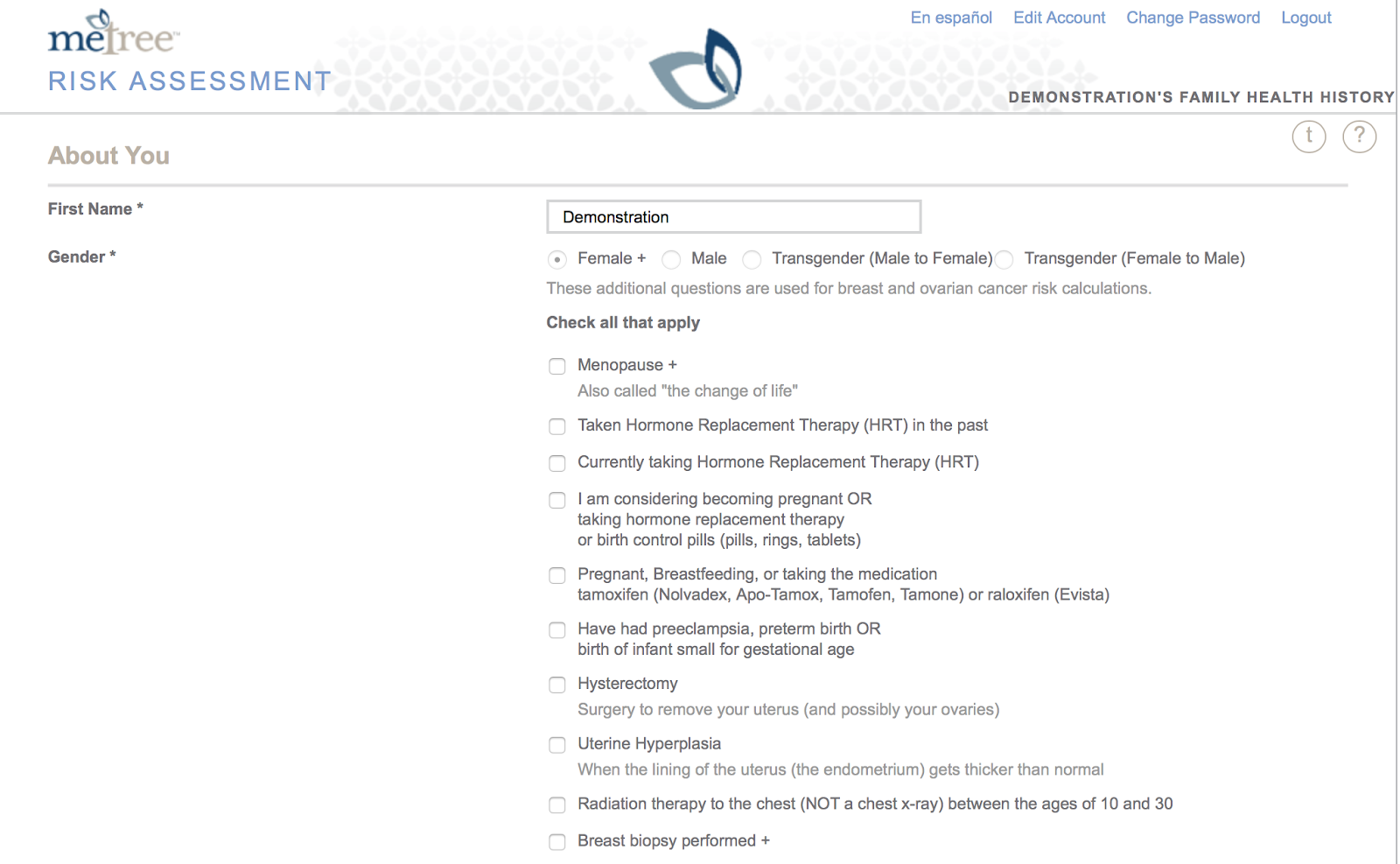


|  |
| --- |

Q5.3
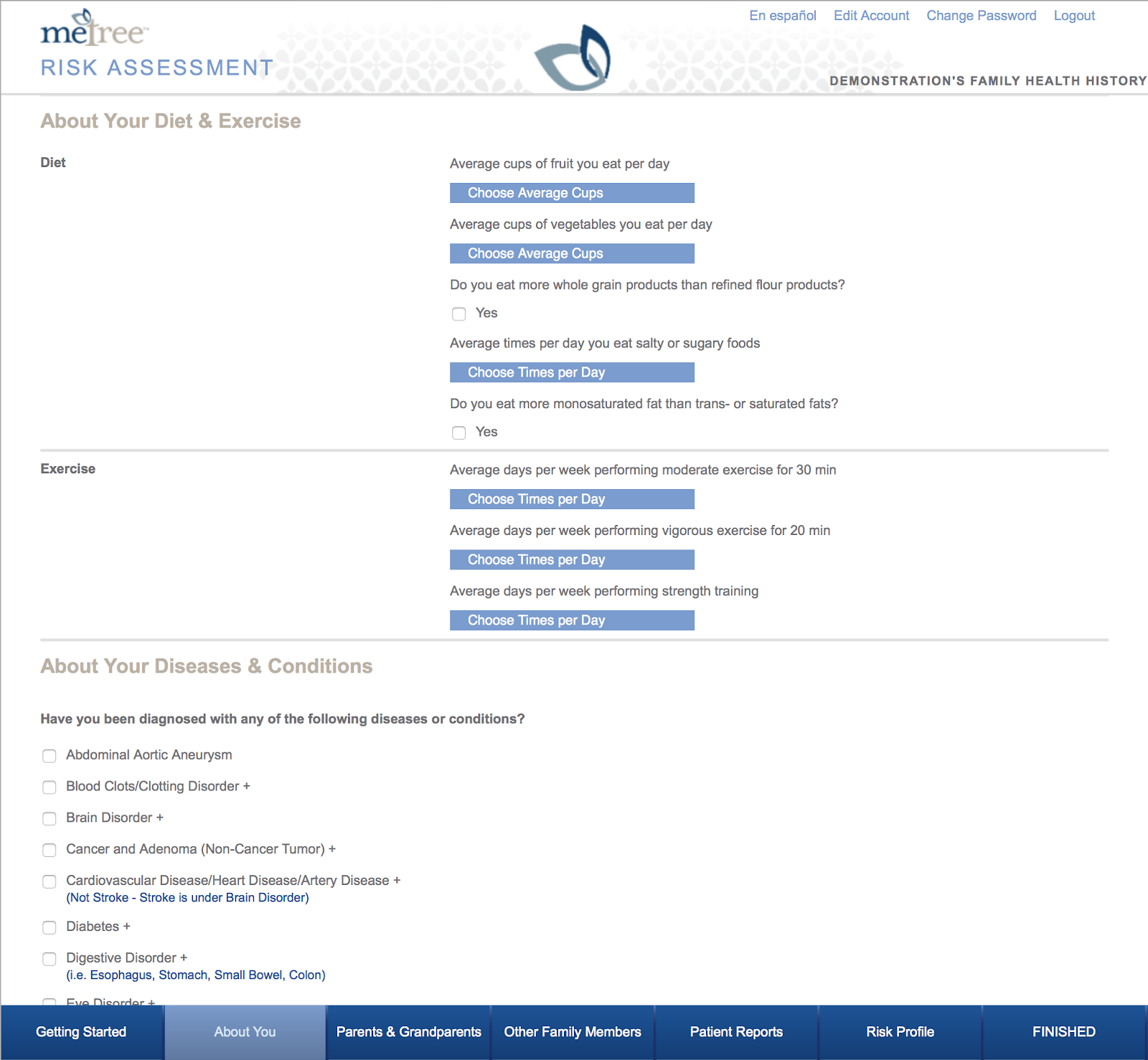


|  |
| --- |

Q5.4


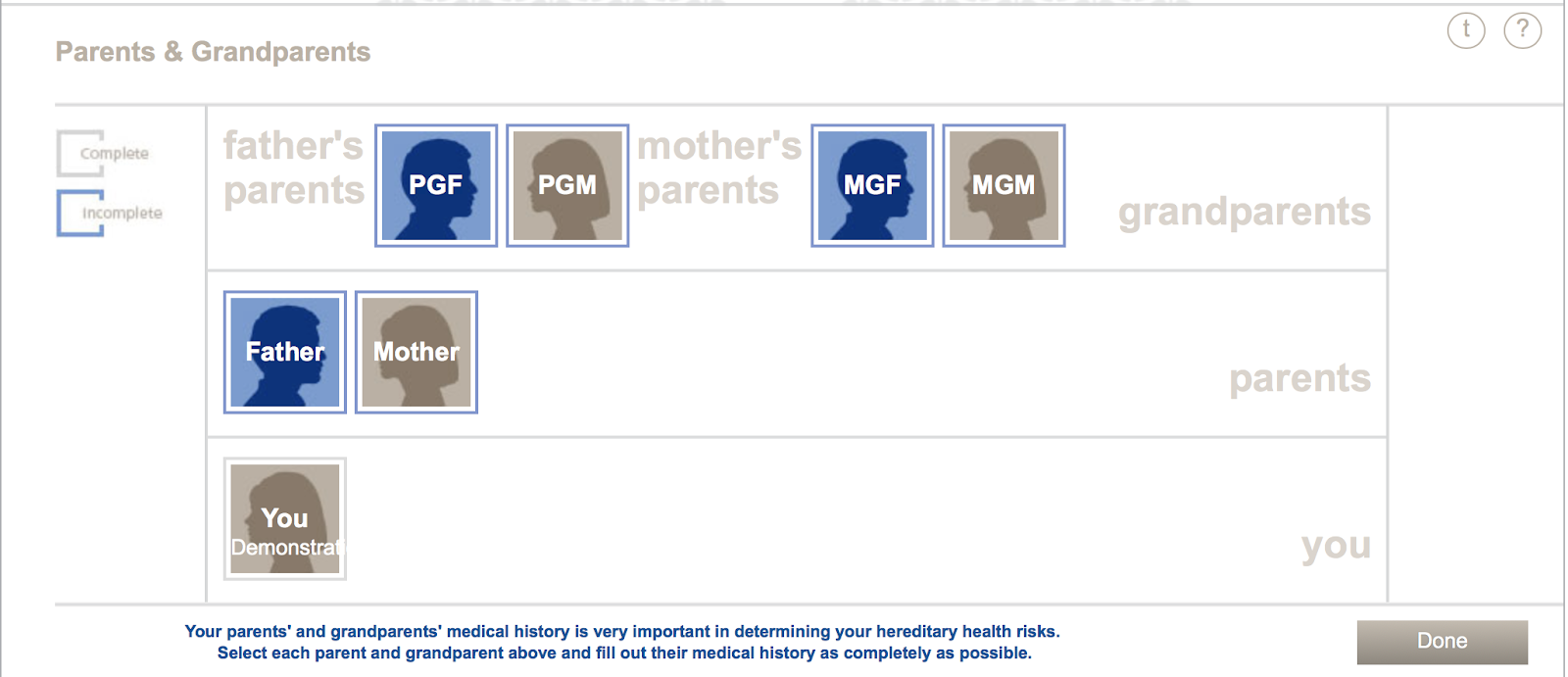


|  |
| --- |

Q5.5


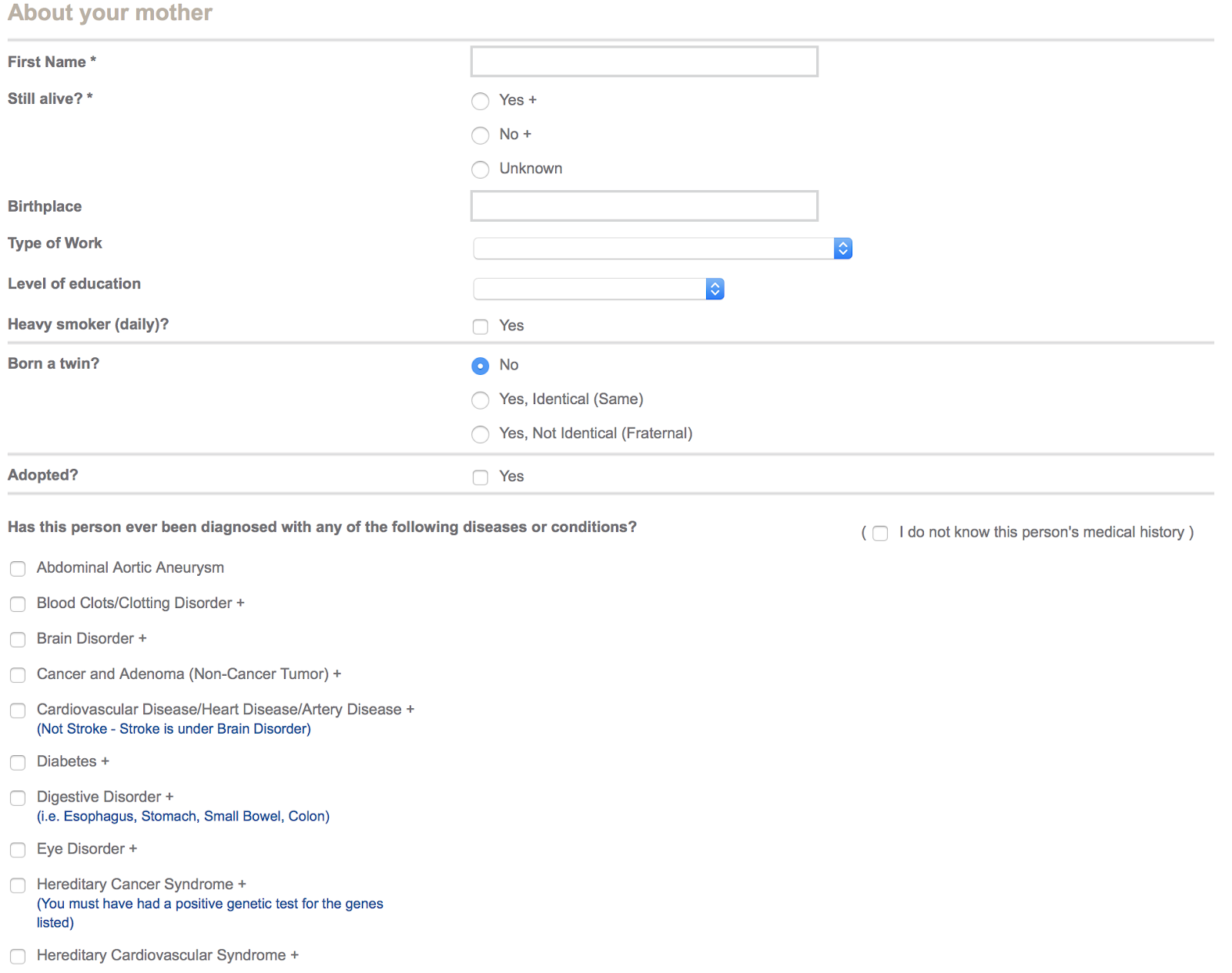


|  |
| --- |

Q62


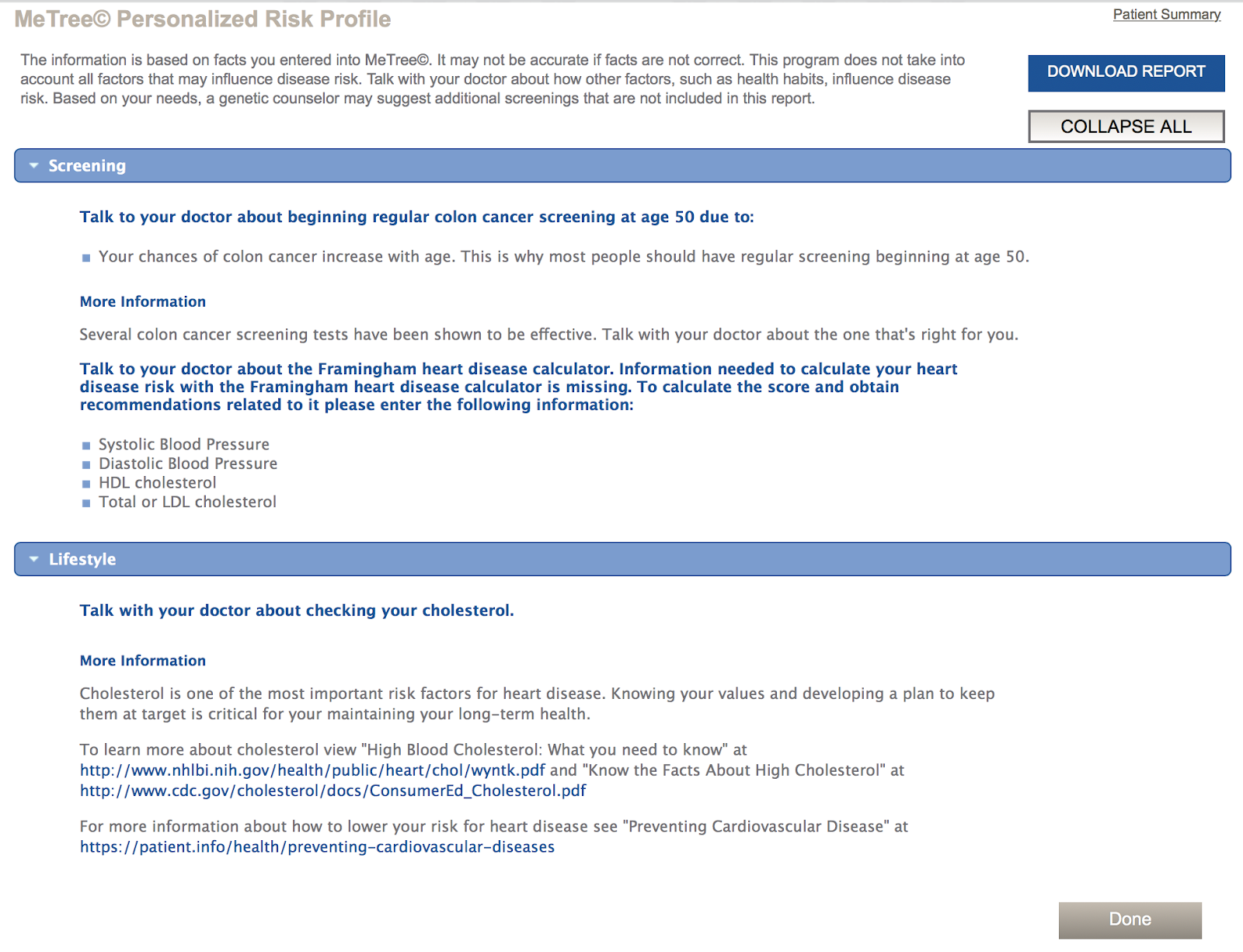


|  |
| --- |

Q5.6 Typical users take around 30 minutes to complete MeTree. After reading the description of MeTree, please rate your responses to the following statements as truthfully as you can.

|  |
| --- |

Q5.7 How comfortable would you be sharing your personal health data with MeTree?

- Very Uncomfortable  (1)
- Somewhat Uncomfortable  (2)
- Somewhat Comfortable  (3)
- Very Comfortable  (4)

|  |
| --- |

Q5.8 How likely is it that you will do the following?

|  | Very Unlikely (1) | Somewhat Unlikely (2) | Somewhat Likely (3) | Very Likely (4) |
| --- | --- | --- | --- | --- |
| Create an account and start a profile on MeTree. (1) |  | • | • | • |
| Complete my profile on MeTree in exchange for a risk report. (2) |  | • | • | • |
| Contact family members to collect health history information so I could fill out MeTree to the best of my knowledge. (3) |  | • | • | • |
| Update information on MeTree in the future when I learn new information about my family health history. (4) |  | • | • | • |
| Recommend MeTree to my family members. (5) |  | • | • | • |
| Recommend MeTree to my friends and colleagues. (6) |  | • | • | • |

|  |
| --- |

Q5.9 Would you like your doctor to introduce tools like MeTree to patients in his/her practice?

- Yes  (1)
- No  (2)

**End of Block: MeTree Evaluation**
