# Supplementary material for: Awareness of family health history in a predominantly young adult population
Source: PLoS One. 2019 Oct 25;14(10):e0224283. doi: 10.1371/journal.pone.0224283 (PMC6814221; doi:10.1371/journal.pone.0224283)
Supplement: S2 Text — (DOCX) [file pone.0224283.s002.docx]

**Written Materials:**

**KNOWING YOUR FAMILY HEALTH HISTORY COULD SAVE YOUR LIFE!**

**Why is my family health history so important?**

**Several important reasons to know your family health history**

- Because some conditions run in families and by asking about your family health history, you can find out what diseases you and your children may be at **increased risk** for getting.
- Depending upon the disease and your risk level (see below) you may meet criteria for more or different kinds of prevention/screening. MeTree calculates your risk from the information you enter and tells you what options are available to lower your risk.
- How much your risk is increased depends upon the type of risk you have.

There are two kinds of **RISK:**

- 1. **Familial risk**. This means you have a slightly higher chance (about 2-3 times higher) of getting the disease than most people outside your family.

An example of a family history for **familial risk** is a mother with colon cancer after age 50.

- 1. **Hereditary risk**. This means you have a much higher chance (**50- 100%**) of getting the disease than most people outside your family. These risks are inherited from a parent through DNA segments (genes) and are not common (only about 1 in 500 people have them).

An example of a family history that might indicate risk for **hereditary risk** is a parent with colon cancer at age 40.

**What does it mean to have a higher risk for cancer?**

**Examples of risk, both Familial Risk and Hereditary Risk**

- Breast cancer occurs in 1 out of 9 women; this means the average person has an **11%** chance of getting breast cancer in their lifetime (called **population risk**). If you have a **familial risk** for breast cancer then you would have a **22-33%** chance of having breast cancer in your lifetime.
- Hereditary risk (risks inherited from a parent through genes), if you have hereditary breast and ovarian cancer syndrome you have an **80%** chance of getting breast cancer in your lifetime.
- The chart below illustrates the risk differences between population risk, familial risk and hereditary risk:
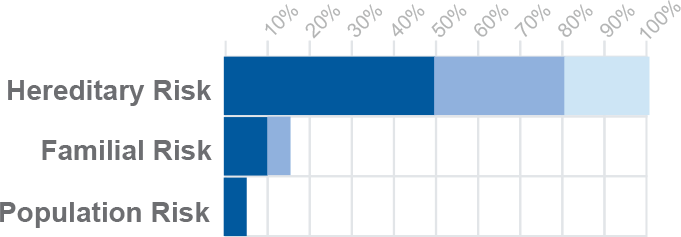


**What exactly is a family health history and what do I ask about?**

**Family members health and how to collect the information**

- A family health history is a record of your blood family members (parents, grandparents, aunts/uncles, brothers/sisters, children, and cousins). Your partner and his/her relatives are not your blood relatives and therefore, their family health history is not helpful in determining your risk
- Record relationship to you (mother, grandmother on mom’s side, etc.), any disease they had, the age they developed the disease, and if they have died -what age they died and what the cause of death was.
- A worksheet with a list of all the conditions you should ask family members about is provided at the end of this document.
- As you saw in the description of familial vs. hereditary risk it is very important to know how old someone was when they developed a disease.
- Important distinctions to make when asking about cancers.
- Most cancers start in one site (site of origin) but often they will spread to other places (metastases). For example lung cancer can frequently spread to the brain.
- When asking about cancer we only need to know the site of origin.

The other sites are not helpful in assessing your risk level.

**How do I ask about family health history?**

**Who to ask and where to start**

- Family members are the best source of information.
  - Start with your parents if they are still alive
  - Often there is on family member who is considered the family historian and knows everything about everyone. If there is a person like this in your family you should talk with him/her.
- Family events like birthdays, Christmas, and weddings are an excellent time to ask, because so many relatives are in one place. In fact, Thanksgiving is a national family health history day and would be a great time to bring it up!

- Some relatives are uncomfortable talking about health and/or medical histories. If that happens to you, you can explain how it will help you and your children

**Why do I also have to provide information about myself?**

**Your health information is also important**

- - To perform a risk assessment for some conditions like hereditary cancer syndromes or hereditary liver diseases you only need family health history to calculate risk; however other diseases need information about yourself and your lifestyle to fully calculate your risk.
  - Examples of these include cholesterol and blood pressure for heart disease risk, radiation exposure for breast cancer risk, and diet for diabetes risk.

**MeTree asks about the following tests for yourself.**

**Check to see if you’ve had any of these tests and what the results were.**

! Cholesterol: *including total, LDL (bad cholesterol), HDL (good cholesterol), and triglycerides (fat)*

! HgbA1c: *a marker of your average sugar levels for the last 3 months, used in diabetes screening and management*

! High sensitivity CRP (hsCRP): *a marker of inflammation used to measure risk of heart disease*

! Carotid Artery Intimal Medial Thickness (CA-IMT): *an ultrasound measuring the thickness of the carotid arteries (it’s not the same as just measuring the blood flow inside the arteries which is called a carotid ultrasound)*

! Coronary Calcium CT Score: *measures calcium deposits in the heart blood Vessels*

! Colonoscopy or Sigmoidoscopy: *used to look inside the colon to find colon cancer*

**MeTree asks about the following family health history.**

**Please talk with family members to see if anyone has been diagnosed with any of these conditions.**

**Types of Cancer:**

! *Adrenal cortex* ! *Liver (or Hepatocellular)*

! *Prostate (Gleason score)*

| ! | *Bone* | ! | *Lung* | ! | *Rectal* |
| --- | --- | --- | --- | --- | --- |
| ! | *Brain* | ! | *Muscle (or Sarcoma)* | ! | *Retinoblastoma* |
| ! | *Breast (both or just one?)* | ! | *Ovarian* | ! | *Skin (which type? more than once?)* |
| ! | *Carcinoid* | ! | *Pancreatic* | ! | *Small Bowel* |
| ! | *Colon (more than once?)* | ! | *Prostate* | ! | *Stomach* |
| ! | *Esophageal* | ! | *Paraganglioma* | ! | *Thyroid (which type)* |
| ! | *Kidney (more than once?)* | ! | *Pheochromocytoma* | ! | *Thyroid nodule (not cancer)* |
| ! | *Leukemia* | ! | *Parathyroid Adenomas* | ! | *Uterine* |
|  |  |  | *(how many?)* |  |  |
| ! | *Lipoma* | ! | *Pituitary Adenoma* |  |  |

**Hereditary Cancer Syndromes (these are uncommon and require a genetic test):**

! *Hereditary Breast & Ovarian Cancer Syndrome*

! *Lynch Syndrome (also called Hereditary Nonpolyposis Colon Cancer)*

! *MUTYH-associated polyposis*

! *Hereditary Diffuse gastric cancer*

! *Juvenile polyposis*

! *Peutz-Jegher’s syndrome*

! *Birt-Hogg-Dube syndrome*

! *Hereditary leiomyomatosis and renal cell carcinoma syndrome*

! *Hereditary papillary renal cancer syndrome*

! *Hereditary retinoblastoma*

! *Malignant hyperthermia*

! *Familial Adenomatous Polyposis*

! *LiFraumeni Syndrome*

! *Cowden Syndrome*

! *Von Hippel-Lindau syndrome*

! *Tuberous sclerosis complex*

! *Hereditary melanoma*

! *Nevoid basal cell carcinoma syndrome*

! *Multiple endocrine neoplasia (MEN) type I*

! *Multiple endocrine neoplasia (MEN) type 2*

! *Hereditary paraganglioma- Pheochromocytoma syndrome*

**Hereditary Cardiovascular Syndromes (these are uncommon and require a specialist for care):**

*! Familial hypercholesterolemia ! Long QT syndrome*

*! Hypertrophic Cardiomyopathy ! Brugada syndrome*

*! Left ventricular non-compaction ! Catecholaminergic polymorphic Syndrome Ventricular tachycardia*

*! Marfans syndrome*

*! Arrhythmogenic right ventricular dysplasia*

*! Ehlers Danlos syndrome*

**Hereditary Liver Diseases:**

_!_ *_Hemochromatosis_* ! *Primary Biliary Cirrhosis*

_!_ *_Wilson’s disease_* ! *Auto-immune  hepatitis*

! *Alpha 1 Anti-trypsinase Deficiency* ^!^ *^Sclerosing  Cholangitis^*

**Hereditary Blood Clotting Diseases:**

_!_ *_Protein C deficiency_* ! *Prothrombin 2010 mutation*

! *Protein S deficiency* ^!^ *^Factor V Leiden^*

! *Anti-thrombin 3 deficiency*

**Other Diseases:**

_!_ *_Aortic Aneurysm_* ! *Multiple Sclerosis*

_!_ *_Asthma_* ! *Obesity*

! *Atrial Fibrillation* ^!^ *^Osteoporosis^*

_!_ *_Blindness_* _!_ *Parkinson’s Disease*

_!_ *_Dementia/Alzheimers_* ! *Peripheral Artery Disease*

_!_ *_Carotid Stenosis_* ! *Rheumatoid Arthritis*

_!_ *_Colon Polyps_* ! *Sickle Cell Trait/Disease*

_!_ *_Crohn’s disease_* ! *Sudden cardiac death*

^!^ *COPD*

! *Diabetes (specify type: type 1, 2, or gestational (pregnancy))*

! *Stroke (specify type- bleeding, low oxygen)*

! *Thyroid Disease*

! *Thalessemia*

! *Glaucoma* ! *Heart Attack*

_!_ *_High Blood Pressure_* ! *Ulcerative Colitis*

_!_ *_High Cholesterol_* ! *Addiction (drugs or alcohol)*

_!_ *_Intellectual disability_* ! *Kidney Disease (specify type:*

! *_Lupus_ nephritis, nephrotic, cystic, diabetes)*

! *_Macrocephaly_* ! *Macular degeneration*

**Mental Illness:**

! *Addiction (drugs or alcohol)* ^!^ *^Obsessive Compulsive^*

! *ADD* ! *Panic/anxiety disorder*

! *Autism* ! *Personality disorder*

! *Bipolar* ! *PTSD*

! *Depression* ! *Schizophrenia*

! *Eating Disorder* ! *Social phobia*

**Tobacco Use**

**Causes of Death:**

_!_ *_Accident_* ! *Natural Causes*

! *Cancer* ^!^ *^SIDS^*

! *Diabetes* ^!^ *^Stroke^*

! *Heart Disease* ^!^ *^Unknown^*

! *Infection* ^!^ *^Other^*

! *Lung Disease (ex. copd)*

**Family History Worksheet**

| **Your Children** | Age now or at death* | Diseases this person has had and age they were diagnosed  Example: Breast Cancer (age 40); blood clots (age 20)  If the person has died, please write cause of death |
| --- | --- | --- |
|  |  |  |
|  |  |  |
|  |  |  |
|  |  |  |
|  |  |  |
|  |  |  |

| **Your Brothers and Sisters** | Age now or at death* | Diseases this person has had and age they were diagnosed  Example: Breast Cancer (age 40); blood clots (age 20)  If the person has died, please write cause of death |
| --- | --- | --- |
|  |  |  |
|  |  |  |
|  |  |  |
|  |  |  |
|  |  |  |
|  |  |  |

| **Your Nieces and Nephews** | Age now or at death* | Diseases this person has had and age they were diagnosed  Example: Breast Cancer (age 40); blood clots (age 20)  If the person has died, please write cause of death |
| --- | --- | --- |
|  |  |  |
|  |  |  |
|  |  |  |
|  |  |  |
|  |  |  |
|  |  |  |

* If you don’t know their exact age, put approximate age.

**Video Intervention:**

Video 1: What is Family Health History? <https://youtu.be/dnhN7Se-I8Y>

Video 2: How can I find my Family Health History? <https://youtu.be/vgAYeBbp9mQ>

Video 3: Addressing Concerns about Family Health History Collection <https://youtu.be/CCJ3AjAO3LY>

Video Intervention Script:

**VIDEO 1: FHH Value (Motivational Intervention)**

*Scene 1: Clinic Office*

Doctor: Welcome to MeTree, a digital tool that can help you and your doctor work together to identify and prevent diseases that run in your family. MeTree collects details about your family member’s health conditions and about your personal health -- including things like diet, exercise, and smoking habits. Much like any form that you fill out when you see a new doctor, MeTree will ask you about your health conditions and any health conditions that run in your family. MeTree uses the information that you enter about you and your family to generate a report that will identify whether you have higher risks of developing specific diseases, like diabetes, heart failure, various cancers, and other rare conditions. MeTree also gives you recommendations for how to talk to your doctor. Talking to your doctor early on about screening tests and treatment options for these diseases has the potential to save your life, and even can save your loved ones’ lives. (0:35)

*Scene 2: Home*

Gary: *(BROLL)* **Sitting at home kitchen table**

*Over screen: “Based on true events.”* When I first filled out MeTree, I wasn’t expecting to find much. I am a generally healthy man, and I hadn’t been diagnosed with any major health problems. After filling out MeTree, MeTree gave me a report **shows report** that suggested I talk to my doctor because I met criteria for breast and prostate cancer screening.

Gary: *(BROLL) *Filling out MeTree** I didn’t even know men could get breast cancer! I knew that my mom and my mom’s mom both had breast cancer and that my dad had prostate cancer when he was older. But, it wasn’t until I inputted this information into MeTree that I realized my parents’ and my grandparents’’ conditions meant something for not just my health, but also my kids’ health. (1:05)

*Scene 3: (BROLL) Clinic Exam Room*Talking with doctor.**

Gary: MeTree sent a report to my doctor. She and I talked about genetic testing for hereditary breast cancer. After getting tested, I found out that the BRCA2 mutation runs in my family, which puts me at an increased risk of breast cancer and prostate cancer. I encouraged my kids, sisters, and brothers to get tested too. Through this process, we found out that two of my kids and my sister also have the harmful mutation (1:26).

*(BROLL) *Sitting in waiting room of doctor’s office with kids**

Gary: Now, I undergo more careful monitoring for breast and prostate cancer regularly to make sure that I’m in good health. If I do develop cancer, I feel more at ease knowing we are more likely to catch it early and take action.

*(BROLL) Scene 4: Park *Playing soccer in park with kids at family gathering**

Gary: While the process was scary at first, I feel so fortunate to have found this information early on. I realize how important sharing family health history was for me and my kids; I feel confident that the process has given my family hope for more happy and healthy years together (1:50-2:00).

**____________________________________________________________________________**

**VIDEO 2: Communication Advice (Communication Self-Efficacy Intervention)**

*Scene 1: Clinic Office *holding tablet**

Doctor: Entering information into MeTree is relatively easy. But sometimes finding the information you need to enter can be difficult. Some of my patients don’t know how or where to find this life-saving information. Here are some suggestions for ways you can find your family health history. (0:15)

- *(“WHO” words transition onto screen):* The best way to find your family health history is to talk to your blood-related parents, aunts, uncles, and even grandparents. Although in-laws or family friends might feel like family, only biological relatives’ history are useful for understanding disease risk. (0:30)
- *(“WHEN” words transition onto screen)*: Family gatherings, like Thanksgiving, weddings, family dinner parties, and holidays can be convenient times for having discussions about family health history. (0:37)
- *(“WHAT” words transition onto screen)*: The most important information to seek includes:
  - Any familial diseases or conditions -- like diabetes or heart attacks
  - The age that your relative received their diagnosis.
    - Gary: For example, I found out that my mom was diagnosed with breast cancer at age 37.
  - If your relative is no longer alive, finding out how old they were when they died and how they died are both very important for understanding your disease risk.
    - Gary: I never met my grandmother, but my mom told me she died at 41 of breast and ovarian cancer. I made sure to provide this information when I was filling out MeTree.
  - Ethnicity, lifestyle habits (like whether they smoked or exercised), and any known allergies for your relatives are also very helpful. (0:47)
    - Gary: I entered into my MeTree that my family and I are Ashkenazi Jewish; I found out that we have much higher risks for certain diseases because of our ancestry! (1:20)

1. *(“HOW” words transition onto screen)*: Here are some conversation tips for asking your family about their family health history. (1:26)

*Scene 2: Woman and Father over dinner with TV in the background*

Anthony: Hey Mom, I know that you have diabetes. My doctor told me that diabetes can run in families and that I might have a higher risk of diabetes, too. Can we talk about your diabetes?

Mother: Sure, Anthony. What do you want to know?

Anthony: Well, when did you first find out you had diabetes?

Mother: I can’t quite remember. But I know I was pretty young. I think that was when we had first moved here. I was about 40. Or maybe I was 42?

Anthony: Do you have any other health problems that you think might run in the family?

Mother: Other than my high sugar, I know your dad and I both also have high blood pressure. Your grandfather also had diabetic problems, but he passed away from lung cancer at 65, as you remember.

Anthony: I remember. Do you remember if grandpa smoked?

Mother: He smoked a pack a day! I never took to the stuff, though.

Anthony: That’s good; I’m glad. What do you do to manage your diabetes?

Mother: I just do my best to exercise and eat healthy. I monitor my blood sugar, but I don’t take insulin or anything. That’s why I’m not eating the pie your dad made! It helps with my high blood pressure *and* the diabetes.

Anthony: That’s really helpful; thanks mom. I’m going to go ask dad a few questions about his health history, now. (~2:30)

*Scene 3: Clinic Office*

Doctor: It’s important to remember that some information is more valuable to your healthcare provider than others. *(Words transition onto screen: “What information matters most?”)*

At a minimum, try to find out what your parents and grandparents have been diagnosed with and when they were diagnosed. If you have family members who have passed, it is helpful to know the age that they passed and the cause of their death.

It is also important to remember to collect accurate information, but don’t worry if your family can’t provide exact information. For example, if you don’t know whether your father was 40 or 42 when he got sick, don’t worry! MeTree will allow you to enter the information within an approximate age-range.

Again, make sure that the family members you are asking about are biologically related or “blood relatives.” **show infographic of family tree** This includes your mother and father, and their siblings. Your spouse; his or her family members; someone who married into the family; or someone who has raised you without blood relation -- like a godparent or adopted parent -- *cannot* provide useful family health history information for the purposes of your medical care, since they don’t share your genes. (~3:40)

*(Words transition onto screen: “Cancers”)*

There are some important distinctions to make when you are asking your family members about cancer. First, various cancers can move and grow, starting at one site. This is called the “site of origin.” But often they can spread, or “metastasize,” to other parts of the body. For example, lung cancer can frequently spread to the brain. *(Words transition onto screen next to “Cancer”: “Site of Origin”)* When MeTree asks about cancer, we only need to know the site of origin. The other sites are not as helpful in assessing your actual risk for cancer. (~4:05)

*(Words transition onto screen: “Family Records”)*

Some families have formal records of family health history, and some families have “family historians” who may be knowledgeable about the health conditions that certain family members had. These resources can be extremely useful when collecting family health history for MeTree.

*(Words transition onto screen: “Family Dynamic”)*

If your family is fairly open to discussing health and disease, you may not even have to wait until the next family gathering to talk about family health history. You could communicate via a phone call, text, or even email.

Some families communicate health differently. Your relatives may prefer to keep their medical history private. In this case, you might consider taking the time to explain to your relatives that sharing their health history is very important for you and your health. Explain to them that sharing their family health history with you may be able to improve your risks for deadly diseases like cancer or heart disease before they develop. After all, knowing your family health history is one of the most powerful ways to reduce your disease risk. Telling your family member the health benefits of family health history might convince them to be more willing to share.

If you are feeling hesitant about asking for your family’s health history, you could potentially ease into the conversation by first discussing other traits that run in your family but are less relevant to health, such as hair color; eye color; or freckles. Discussing how certain physical traits are inherited might make it easier for your relatives to understand how *their* health history is important for *your* health.

*(Words transition onto screen: “Conditions to Ask About”)*

Make an effort to ask about diseases that tend to run in families. Examples of other diseases that run in families include … (~5:15)

*(Words scroll along screen as doctor reads them aloud)*

- Cancer
- High cholesterol
- Anemias
- Asthma
- Chronic Obstructive Pulmonary Disease
- Crohn’s disease
- Glaucoma
- Lupus
- Multiple sclerosis
- Obesity
- Parkinson’s disease
- Anxiety
- Stroke
- Heart attack
- Schizophrenia

Doctor: We have provided worksheet to help guide your collection of this information.

**____________________________________________________________________________**

**VIDEO 3: FHH and MeTree Concerns (Patient Empowerment Intervention)**

*Scene 1: Clinic Office*

Doctor: The idea of collecting family health history and inputting your personal information into a tool like MeTree might cause some patients to experience feelings of stress or anxiety. In this video, we will address some of the concerns that patients may have before they fill out MeTree: (0:13)

*Scene 2: Clinic Room. Patient sitting on examination table.*

Patient: I’m worried about entering my family health information for a couple reasons. First, I’m concerned that others will be able to find out about my family’s health information.

Doctor: Your family health history is protected under the same federal laws that protect all your other health records. This law, called the Health Insurance Portability and Accountability Act -- or HIPAA -- guarantees that the information you enter into MeTree will be kept private.

Patient: I’m also concerned that learning about my family health information will make me feel more anxious, or force me to get future tests that I don’t want.

Doctor: Those are valid concerns. Some patients feel anxious before filling out MeTree; these patients might be worried to find out they are at an increased risk for a certain disease and that this discovery will cause unnecessary or overwhelming stress. Some patients think they are better off if they don’t know their risks for a disease in advance.

This is understandable. Luckily however, studies repeatedly show that patients who share their family health history with their clinician feel more satisfied with their healthcare because they know more about their risks for disease and can do more to manage these risks. Studies also show that even patients who do discover their increased risks for disease do not usually experience psychological distress. Instead, these patients are often empowered to make better health decisions that will improve their health outcomes in the long run. (~1:25-1:30)

**Patient holds report, doctor holds report*/ zoom to patient decoy report*

When MeTree generates a report, it lists some of the diseases you might be at a higher risk for and gives you suggestions for what you can do. While MeTree’s personalized recommendations aim to improve patient outcomes, you do not have to follow any of MeTree’s suggestions based on your health history if you don’t want to. It is important to remember that while your doctor has your best interests in mind, it is up to you to communicate your preferences about any potential next steps. Completing the MeTree report before your scheduled appointment can help you prepare for a conversation with your doctor about any of your concerns and what *you* want and don’t want when managing your health risks. (1:55)

Patient: What if my mother does not want to find out about her family health history? Won’t this MeTree report force her to know things she doesn’t want to know?

Doctor: You are the only person who will see your final MeTree report. Your family members will not receive a copy. So, you can share this information with her or other family members at your discretion. The MeTree report is meant to help you and your family members, including your kids, make the best health decisions based on your family health history. Although sharing this information with your family members may help them understand their health risks, you have the option to keep the results private. (2:20)
