# Supplementary material for: Awareness of family health history in a predominantly young adult population
Source: PLoS One. 2019 Oct 25;14(10):e0224283. doi: 10.1371/journal.pone.0224283 (PMC6814221; doi:10.1371/journal.pone.0224283)
Supplement: S1 Table — (DOCX) [file pone.0224283.s003.docx]

**S2 Table. A comparison of the features of the video and written MeTree© educational materials.**

| **Feature** | **Written** | **Narrative Video** |
| --- | --- | --- |
| Reading level^1^ (Flesch Reading Ease Score, Flesch-Kincaid Grade) | 52.2 (8th Grade) | 56.4 (9th Grade) |
| Word Count | 1618 | 2078 |
| Graphs | Yes | No |
| Detailed description of FHH information needed | Yes | Yes |
| Detailed distinction between “relatives” via matrimony vs. birth | Yes | Yes |
| Detailed distinction between sociocultural family and biological family (e.g. godmother vs. mother) | No | Yes |
| Defines familial vs. hereditary risk terminology | Yes | No |
| Action-oriented statements, instructions | Yes | Yes |
| Q&A-style discussion | Yes | Yes |
| Suggestions for potential FHH collection strategies | Yes | Yes |
| Examples, models for family conversation | No | Yes |
| List of diseases to ask about | Comprehensive | Recognizable list of 18 rare and hereditary diseases |
| Includes layman’s name for conditions (e.g. “high sugar” for diabetes) | No | Yes |
| Example Patient Narrative/Experience | No | Yes |
| Response to common patient concerns about privacy, health records | No | Yes |
| Response to common patient concerns about potential anxiety from disease screening results | No | Yes |
| Description of MeTree inputs | Yes | Yes |
| Description of MeTree outputs | No | Yes |
| Subsections | 8 distinct sub-headers | 3 videos |
| Worksheet included for FHH collection | Yes | No |
